# Dominant Species in Subtropical Forests Could Decrease Photosynthetic N Allocation to Carboxylation and Bioenergetics and Enhance Leaf Construction Costs during Forest Succession

**DOI:** 10.3389/fpls.2018.00117

**Published:** 2018-02-08

**Authors:** Yihua Xiao, Shirong Liu, Fuchun Tong, Bufeng Chen, Yuanwen Kuang

**Affiliations:** ^1^Research Institute of Tropical Forestry, Chinese Academy of Forestry, Guangzhou, China; ^2^Key Laboratory of Forest Ecology and Environment, China's State Forestry Administration, Institute of Forest Ecology, Environment and Protection, Chinese Academy of Forestry, Beijing, China; ^3^College of Forestry and Landscape Architecture, South China Agricultural University, Guangzhou, China; ^4^Key Laboratory of Vegetation Restoration and Management of Degraded Ecosystems, South China Botanical Garden, Chinese Academy of Sciences, Guangzhou, China; ^5^Guangdong Provincial Key Laboratory of Applied Botany, South China Botanical Garden, Chinese Academy of Sciences, Guangzhou, China

**Keywords:** leaf construction cost, photosynthesis, nitrogen allocation, specific leaf area, forest succession

## Abstract

It is important to understand how eco-physiological characteristics shift in forests when elucidating the mechanisms underlying species replacement and the process of succession and stabilization. In this study, the dominant species at three typical successional stages (early-, mid-, and late-succession) in the subtropical forests of China were selected. At each stage, we compared the leaf construction costs (CC), payback time (PBT), leaf area based N content (*N*_A_), maximum CO_2_ assimilation rate (*P*_max_), specific leaf area (SLA), photosynthetic nitrogen use efficiency (PNUE), and leaf N allocated to carboxylation (*N*_C_), and to bioenergetics (*N*_B_). The relationships between these leaf functional traits were also determined. The results showed that the early-succession forest is characterized with significantly lower leaf CC, PBT, *N*_A_, but higher *P*_max_, SLA, PNUE, *N*_C_, and *N*_B_, in relation to the late-succession forest. From the early- to the late-succession forests, the relationship between *P*_max_ and leaf CC strengthened, whereas the relationships between *N*_B_, *N*_C_, PNUE, and leaf CC weakened. Thus, the dominant species are able to decrease the allocation of the photosynthetic N fraction to carboxylation and bioenergetics during forest succession. The shift in these leaf functional traits and their linkages might represent a fundamental physiological mechanism that occurs during forest succession and stabilization.

## Introduction

Understanding plant community succession is a fundamental objective of ecology (Buma et al., 2017). With the development of sophisticated experimental techniques (Castle et al., 2016), research on community succession has explored the commonalities and differences in the assemblages of different plant communities, particularly in association with climate change (Pearson et al., 2013). As a result, we are expanding our conceptual and quantitative understanding of many aspects of plant community succession (Walker and Wardle, 2014; Meiners et al., 2015).

There are clear differences in the composition of dominant species between severely disturbed forests and intact forests, because of differences in light density, air humidity, temperature, nutrient availability, and soil processes (Wardle et al., 2004; Prach and Walker, 2011). How forests convert from one form to the other could be tracked using a variety of plant functional traits (Raevel et al., 2012) that have considerable ecological and evolutionary impacts on terrestrial ecosystems (Moran et al., 2014; Zeppel et al., 2014; Knapp et al., 2015). Therefore, many of the functional traits could, individually or in combination, indicate how plants respond to environmental change (Pratt and Mooney, 2013; Rillig et al., 2015). Examples of such traits include leaf construction costs (hereafter, leaf CC; Poorter and Evans, 1998; Pýankov et al., 2011; Falcão et al., 2015, 2017; Kunstler et al., 2016), leaf morphology, stoichiometry, and physiology (Raevel et al., 2012; Böhnke et al., 2014; Chai et al., 2016), and leaf photosynthetic characteristics (Feng et al., 2007; Hikosaka, 2014). Consequently, these traits could provide the potential to explore some of the adaptive strategies of plants for resource acquisition, growth, competition, survival, and mechanistic resistance. Furthermore, these traits could be used to explore the dynamics of plant communities and the processes of forest ecosystems (Picotte et al., 2009; Navas et al., 2010; Pýankov et al., 2011; Raevel et al., 2012; Boukili and Chazdon, 2017).

Leaf CC is a quantifiable way of determining the energy invested by plants to synthesize carbon skeletons and nitrogenous compounds. This trait has frequently been used to evaluate the resistance of plants to abiotic stress (Chai et al., 2015; Falcão et al., 2015, 2017), as well as to reflect the specific growth characteristics and distribution strategies of plants (Feng et al., 2008), and to compare leaf resource use efficiency among plants (Pýankov et al., 2011). Leaf nitrogen (N) determines plant growth potential. In general, over half of the total leaf N is used for photosynthesis (Poorter and Evans, 1998). The partitioning of photosynthetic N among different photosynthetic apparatus (carboxylation, bioenergetics, and light-harvesting components) is positively related to photosynthetic capacity (Onoda et al., 2004, 2017) and strongly influences photosynthetic N use efficiency (PNUE) (Feng et al., 2007; Hikosaka, 2014). It is speculated that species with lower leaf CC and higher specific leaf area (SLA) allocate a higher fraction of leaf N to photosynthetic apparatus and have higher PNUE than the species with higher leaf CC and lower SLA (Warren et al., 2006). However, research remains limited on how dominant species at different successional stages in forests differ in terms of leaf CC, N allocation, and PNUE. Understanding the linkages of leaf CC, N allocation, and PNUE in the dominant species from different succession stages could provide insights on how dominant species survive, in addition to revealing the mechanism of forest succession and stabilization (McGill et al., 2006; Chai et al., 2015).

The Dinghushan Biosphere Reserve in southern China has unique subtropical monsoon evergreen broadleaf forests in the latitudinal zonality where a large area is covered by deserts (Kong et al., 1993). Owing to hundreds of years of anthropogenic disturbance (including logging and biofuel harvesting; Tang et al., 2006), subtropical monsoon evergreen forests have been severely degraded into large number of fragments representing early-, mid-, and late-succession stages in this region (Zhu et al., 2013). Although plant species at different successional stages might experience noticeably different nutrient supply and survival conditions caused by disturbance (Zhu et al., 2013; Ostertag et al., 2015), knowledge remains limited about how these species invest resources and energy, e.g., the leaf CC and the payback time (PBT, the time required for a leaf to fix the same amount of glucose as required to construct that leaf, Karagatzides and Ellison, 2009), and allocate leaf N in photosynthesis. In this study, we selected the dominant species from early-, mid-, and late-succession forests, and compared their leaf functional traits to explore how these traits are linked among the different successional forests. We hypothesized that dominant species would adjust how leaf functional traits are linked [e.g., leaf CC, PNUE, maximum CO_2_ assimilation rate (*P*_max_), N allocation, PBT] as adaptive strategies among the stages of succession.

## Materials and methods

### Study site and forests

This study was conducted in Dinghushan National Nature Reserve (112°30′-112°33′E, 23°09′-23°11′N, 1,133 ha), which is located in the middle part of Guangdong Province in southern China. This reserve has a typical monsoon climate, and is located in a low subtropical humid forest zone (Mo et al., 2006). The mean annual temperature, precipitation, and relative humidity are 20.9°C, 1,956 mm, and 81.5%, respectively, based on the past three decades. There is a distinct dry season (from October to March) and a distinct wet season (from April to September), when ~70% of the precipitation occurs. The lowest monthly mean temperature is 12.6°C in January and the highest is 28.0°C in July (Zhu et al., 2013).

Within this reserve, there are three types of forests representing different stages of forest succession: the monsoon evergreen broadleaf forest (BF), the mixed pine and broadleaf forest (MF), and the pine forest (PF). The BF is the most mature forest type of the three successional stages, and is located at ~250–300 m above sea level, covering ~600 ha. Vegetation in the BF is typical of the low subtropical zone, and has been protected from human impacts for more than 400 years (Zhou et al., 2006). The dominant species are *Castanopsis chinensis, Cryptocarya chinensis, Machilus chinensis, Schima superba*, and *Syzygium rehderianum* in the crown layer. These species account for over 65% of the total standing biomass in the BF (Huang et al., 2013). This forest type is representative of the late-succession forest. The MF formed from a pine forest that was cultured in the 1930s' plantation effort. After 90 years of succession, the plantation is currently characterized as a mid-successional forest dominated by *Aporosa dioica, C. chinensis, Pinus massoniana, S. superba*, and *Schefflera heptaphylla*. These species, combined, account for approximately 90% of total standing biomass. The PF formed 60 years ago after clear-cutting, and is representative of an early-succession forest. *P. massoniana* is the dominant species, in addition to some broadleaf species, such as *A. dioica, Castanopsis fissa, Evodia lepta*, and *S. superba*. *P. massoniana* accounts for over 95% of the total standing biomass. In 2013, three forest stands covering 3,000, 10,000, and 10,000 m^2^, representing the early-, mid- and late-succession forests, respectively, were established in this reserve. Within each forest type, the dominant tree species were selected for leaf sampling (Table 1).

**Table 1:** Dominant species in the pine forest (PF), mixed forest (MF), and evergreen broadleaf forest (BF) of Dinghushan Biosphere Reserve.

**PF**		**MF**		**BF**	
**Species**	**Code**	**Species**	**Code**	**Species**	**Code**
*Aporosa dioica*	Apd	*Acronychia pedunculata*	Acp	*Acmena acuminatissima*	Aca
*Castanopsis fissa*	Caf	*A. dioica*	Apd	*Aidia canthioides*	Aid
*Evodia lepta*	Evl	*Ardisia quinquegona*	Arq	*Aporusa yunnanensis*	Apy
*Mallotus paniculatus*	Map	*C. chinensis*	Cac	*Artocarpus styracifolius*	Ars
*Pinus massoniana*	Pim	*P. massoniana*	Pim	*Bridelia insulana*	Bri
*Schima superba*	Scs	*Psychotria asiatica*	Psa	*C. chinensis*	Cac
		*Schefflera heptaphylla*	Sch	*Chrysophyllum lanceolatum*	Chl
		*S. superba*	Scs	*Cryptocarya chinensis*	Crc
				*Diospyros morrisiana*	Dim
				*Engelhardtia roxburghiana*	Enr
				*Gironniera subaequalis*	Gis
				*Lindera chunii*	Lic
				*Machilus breviflora*	Mab
				*M. chinensis*	Mac
				*Memecylon ligustrifolium*	Mel
				*Microdesmis caseariifolia*	Mic
				*Ormosia glaberrima*	Org
				*Pithecellobium lucidum*	Pil
				*Pygeum topengii*	Pyt
				*S. superba*	Scs
				*Semiliquidambar cathayensis*	Sec
				*Sterculia lanceolata*	Stl
				*Syzygium levinei*	Sym
				*S. rehderianum*	Syr
				*Xanthophyllum hainanense*	Xah

### Photosynthetic measurement

Considering the significant effect of water availability on plant leaf CC (Falcão et al., 2017), we performed the photosynthetic measurement in August 2016, which is the growing season with ~270 mm rainfall, ~740 MJ/m^2^ photosynthetically active radiation (PAR), ~63.0% water content in fresh leaves, 30.4, 32.4, and 22.4% soil moisture (0–15 cm depth) for BF, MF and PF, respectively, in this reserve (Zhang, 2011). The photosynthetic light responses of the selected dominant trees were measured between 08:00 and 11:30 on consecutive sunny days using a portable photosynthesis system (Li-6400XT, Biosciences, Lincoln, NE, USA) with a LED red/blue light source. The photosynthetic photon flux density (PPFD) was set at 1,500, 1,200, 1,000, 800, 500, 300, 200, 100, 80, 60, 50, 20, and 0 μmol·m^−2^·s^−1^, respectively. Ambient conditions were 400 μmol·mol^−1^ CO_2_, 55–65% air humidity, 30°C leaf temperature, and 1.5 kPa vapor pressure.

For each species, at least seven individual trees from the open canopy of each forest were selected for leaf sampling. For each tree, seven fully expanded and undamaged leaves outside the crown with full exposure were selected for the photosynthetic measurement. To minimize the influence of leaf age on the photosynthesis, all leaves were sampled from the current-yearly grown (1 year's old) ones on the outer crown. Afterwards, photosynthetic responses to intercellular CO_2_ concentration (C_i_) was determined under saturated PPFD. The net photosynthetic rate (*P*_n_) was determined at 400, 300, 260, 200, 180, 150, 120, 100, 80, 60, 50, and 20 μmol·mol^−1^ CO_2_ in the reference chamber. Before the measurement, the leaves were exposed to saturated PPFD provided by the LED light source of the equipment for 30 min to achieve full photosynthetic induction. The light saturated photosynthetic rate (*P*_max_) was obtained at 1,500 μmol·m^−2^·s^−1^ light intensity with ambient CO_2_ concentration and atmospheric humidity. Stomatal conductance (G_s_, mol·m^−2^·s^−1^) and intercellular concentration of CO_2_ (C_i_, μmol·mol^−1^) were gained during the measurement. No photoinhibition occurred during the measurements. All the photosynthetic measurements were completed within 15 days in August.

### Chemical measurements

After the photosynthetic measurement, the leaves (needles) were collected and divided into two parts for chemical analysis. One part was used to measure chlorophyll content (Chl) and the other part was used to measure the leaf area. To measure chlorophyll content, two circular leaf disks (diameter 8 mm) were punched from the center of the blade, avoiding the main vein, and dipped in 5 mL 80% acetone to determine the Chl content (Kuang et al., 2017). The Chl a (ug·mg^−1^, fresh weight) and Chl b content was determined by recording absorption at 663 and 645 nm, respectively, using a spectrophotometer (Unico, Shanghai, China). The sum of Chl a and Chl b was defined as the total Chl. The area of broadleaves was measured with a leaf area meter (CI-203; CID Bio-Science, Inc., Camas, WA, USA), whereas the area of individual needles was estimated by measuring 10 needles with a WinFOLIA scanner (LC4800P, Regent, QC, Canada) and WinNEEDLE software. All leaves (broadleaf and needles) were then oven-dried at 65°C to a constant weight to determine the dry mass. The special leaf area (SLA, cm^2^·g^−1^) was calculated as leaf area per dry mass (Feng et al., 2008). The dried leaves (broadleaf and needles) were ground and homogenized for subsequent chemical analysis.

The total leaf N content per unit mass (*N*_M_) was measured with a CHNS-Autoanalyzer (Elementar Vario EL-III, Elementar Analysensysteme GmbH, Hanau, Germany). The total leaf N content per unit area (*N*_A_) was calculated as the product of *N*_M_ and SLA (Zhu et al., 2012). Photosynthetic N use efficiency (PNUE) was defined as *P*_max_ divided by *N*_A_. Leaf ash content (Ash) was measured by burning dry leaf powder for 4 h at 550°C. The heat of combustion (HC) was measured by burning ~1 g of leaf powder with an oxygen bomb calorimeter (OR2012, Shanghai, China). The ash-free heat of combustion was calculated based on HC and Ash (Zhu et al., 2013). For both the Ash and HC measurements, septuplicate samples were analyzed and averaged for each species.

### Construction costs and payback time

The leaf CC was calculated from the growth efficiency of the leaf tissue, HC, Ash, and *N*_M_ (Williams et al., 1987):

$$\text{CC}=\frac{(0.06968HC-0.065)(1-Ash)+7.5\left(\frac{kN_{M}}{14.0067}\right)}{0.89}$$

where HC is the ash-free heat of combustion (kJ·g^−1^); Ash is the ash content (g·g^−1^ leaf dry mass); N_M_ is the total N concentration (g·g^−1^ dry mass); and k is the oxidation state of the N source (+5 for nitrate, −3 for ammonium). We used *k* = −3 because ammonium is the main source of soil nitrogen at the study site (Zhang, 2011).

Payback time (PBT) was calculated as CC_mass_/*P*_mass_ after converting CC_mass_ from g·glucose·g^−1^ dry mass to nmol·g^−1^ dry mass and P_mass_ from μmol CO_2_ g^−1^ dry mass·s^−1^ to nmol C·g^−1^ dry mass·h^−1^ (Karagatzides and Ellison, 2009). In this study, PBT was determined by hour, rather than day, because the diurnal radiation period changes during the growing season. Therefore, PBT in this study represented the theoretically minimum amortization (Shipley et al., 2006).

### Calculations of P_n_-C_i_ curve-related variables

The P_n_-C_i_ curve was fitted with a linear equation (*P*_*n*_ = kC_i_ + i) within 50–200 μmol·mol^−1^ C_i_. Maximum carboxylation rate (*V*_*c*__max_) and dark respiration rate (*R*_d_) were calculated according to Farquhar and Sharkey (1982):

(1) $$\begin{array}{ccc} V_{c\max} & = & k{\left[C_{i}+K_{c}(1+O/K_{o})\right]}^{2}/[\Gamma^{*}+K_{c}(1+O/K_{o})] \end{array}$$

(2) $$\begin{array}{l} \begin{array}{ccc} R_{d} & = & V_{c\max}(C_{i}-\Gamma^{*})/[C_{i}\;+K_{c}(1+O/K_{c})] \end{array}\\ \;\begin{array}{c} -(KC_{i}+i) \end{array} \end{array}$$

where *K*_c_ and *K*_o_ were the Michaelis–Menten constants of Rubisco for carboxylation and oxidation, respectively; Γ^*^ was the CO_2_ compensation point; and *O* was the intercellular oxygen concentration, close to 210 mmol mol^−1^. The values of *K*_c_, *K*_o_, and Γ^*^ were temperature-dependent (Bernacchi et al., 2001). Maximum electron transport rate (*J*_*max*_) was calculated according to Loustau et al. (1999):

(3) $$J_{\max}=\;\left[4\left({P^{\prime }}_{\max}+R_{d}\right)\left(C_{i}+2\Gamma^{*}\right)\right]/\left(C_{i}-\Gamma^{*}\right)$$

The fractions of total leaf N allocated to carboxylation (P_C_, g·g^−1^), bioenergetics (P_B_, g·g^−1^), and light-harvesting components (P_L_, g·g^−1^) of the photosynthetic apparatus were calculated as:

(4) $$P_{C}=V_{c\max}/\left(6.25V_{cr}N_{A}\right)$$

(5) $$P_{B}=J_{\max}/\left(8.06J_{mc}N_{A}\right)$$

(6) $$P_{L}=C_{C}/\left(N_{M}C_{B}\right)\;$$

where C_C_ is leaf Chl content; *N*_M_ is mass-based leaf N content; and *V*_*cr*_ and *J*_*mc*_ are the specific activities of Rubisco (μmol CO_2_ g^−1^ Rubisco s^−1^) and cyt f (mol electrons mol^−1^ cyt f s^−1^), respectively (Niinemets and Tenhunen, 1997). The fractions of leaf N allocated to both carboxylation and bioenergetics (*P*_*C*+*B*_, g·g^−1^), plus all components of the photosynthetic apparatus (*P*_*T*_, g·g^−1^), were calculated as the sum of *P*_*C*_ and *P*_*B*_ and the sum of *P*_*C*_, *P*_*B*_, and *P*_*L*_, respectively. N content in carboxylation (*N*_*C*_), bioenergetics(*N*_*B*_), bioenergetics and carboxylation (*N*_*B*+*C*_), light-harvesting components (*N*_*L*_), and all components of the photosynthetic apparatus (*N*_*P*_) were calculated as the products of *N*_A_ and *P*_*C*_, *P*_*B*_, *P*_*C*+*B*_, *P*_*L*_, and *P*_*T*_, respectively. The fractions of photosynthetic N partitioned to carboxylation, bioenergetics, and light-harvesting components were indicated by *N*_*C*_/*N*_*P*_, *N*_*B*_/*N*_*P*_, and *N*_*L*_/*N*_*P*_, respectively. The photosynthetic-use efficiency of photosynthetic N was indicated by *P*_max_/*N*_P_.

### Statistical analysis

All statistical analyses were conducted using SPSS 17.0 (SPSS software Inc., Chicago, USA). All data of the dominant species within each successional forest were tested for normality and homoscedasticity, and where necessary, the data were log_10_-transformed before analysis. The results are presented as mean ± standard deviation (SD).

Differences among the successional stages were detected using one-way ANOVA. When the differences were significant, the Student–Newman–Keuls test (S-N-K test) at a 5% probability was conducted. Relationships among the leaf functional traits within each successional stage were tested using linear regression analysis. Pearson's correlation coefficient was used to evaluate the degree of relationship between the functional traits.

Multivariate associations of the leaf functional traits were analyzed by principal component analysis (PCA). The mean values of the traits used were log_10_-transformed. Average factor loading values of different successional species on the first two PC axes were also compared to examine whether these groups were significantly separated along PC axes. Species was used as a fixed factor and variables (indicated by the y-axis and x-axis in each panel) were used as dependent variables and covariates, respectively.

## Results

### Leaf CC and PBT

**Figure 1:**
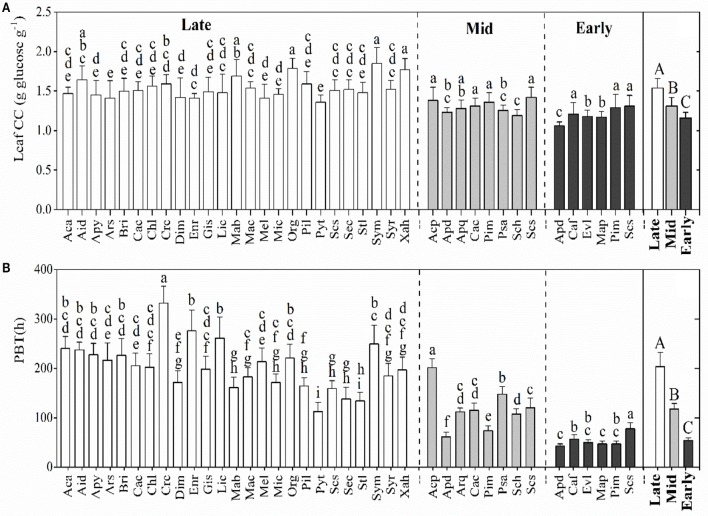
Comparison of leaf construction costs (CC, **A**) and payback time (PBT, **B**) (mean ± SD) of the dominant species within and among the three successional stages. Black, gray, and white bars represent the species from early-, mid-, and late-successional forests, respectively. Differences within or between successional stages were detected by One-Way ANOVA and followed by a Student Newman-Keuls (S-N-K) test if significances exist (*p* < 0.05). Different capital letters denote significant differences among successional stages, and lowercase letters denote significant differences between species within the same successional stage (*p* < 0.05).

The leaf CC of the dominant species significantly varied among the three successional forests (*p* < 0.001), with average values for species ranging from 1.06 to 1.85 g glucose g^−1^. As expected, leaf CC increased with the forest succession, with the highest mean being recorded for the late-succession forest (BF, Figure 1A). Like leaf CC, leaf PBT increased along the successional stages, from 42.76 h in the early-successional forest (PF) to 332.05 h in the late-successional forest (BF, Figure 1B). Both the leaf CC and the PBT were significantly different among some of the species within each successional stage (Figure 1).

### Photosynthetic characteristics and special leaf area

**Figure 2:**
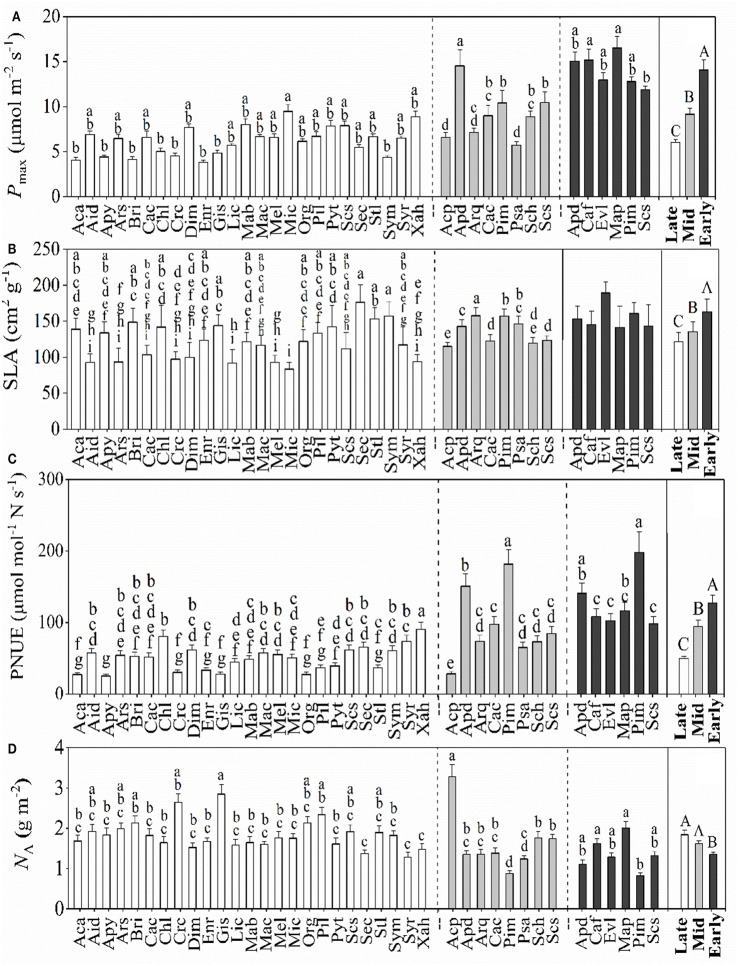
Mean values (±SD) of maximum CO_2_ assimilation rate (*P*_max_, **A**), specific leaf area (SLA, **B**), photosynthetic nitrogen-use efficiencies (PNUE, **C**), and area-based leaf nitrogen (*N*_A_, **D**) for the dominant species in the three successional forests. Black, gray, and white bars represent species from the early-, mid-, and late-succession stages, respectively. Differences within or between successional stages were detected by One-Way ANOVA and followed by S-N-K test if significances exist (*p* < 0.05). Different capital letters denote significant differences among successional stages, and lowercase letters denote significant differences between species within the same successional stage (*p* < 0.05).

The dominant species among the successional stages showed significant differences in *P*_max_, *J*_max_, and *V*_cmax_, Gs and decreased from PF to BF (Figure 2A, Table 2). Conversely, dominant species among the successional stages expressed significant differences in C_i_ with an increasing trend from PF to BF (Table 2). The patterns of leaf gas exchange indicated that the photosynthetic capacity of the dominant species decreased from BF to PF. The specific leaf area (SLA, Figure 2B) and the PNUE (Figure 2C) of the dominant species followed a similar trend to *P*_max_ along the forest successions. Among some of the species within each successional forest, there existed significant differences in *P*_max_, PNUE, and *N*_A_ (Figure 2).

**Table 2:** Differences of the tested variables in the dominant species from the early-, mid-, and late-succession forests (PF, MF, and BF, respectively, mean ± SD) at Dinghushan Biosphere Reserve, southern China.

**Variable**	**PF**	**MF**	**BF**
*J*_max_ (μmol m^−2^ s^−1^)	98.1 ± 11.7a	87.3 ± 9.2b	75.6 ± 8.8c
*V*_cmax_ (μmol m^−2^ s^−1^)	52.77 ± 6.2a	44.97 ± 4.5b	36.29 ± 3.7c
AQY (mol mol^−1^)	0.047 ± 0.006a	0.049 ± 0.005a	0.037 ± 0.04b
*P*_B_ (g g^−1^)	0.06 ± 0.007a	0.05 ± 0.004a	0.03 ± 0.004b
*P*_C_ (g g^−1^)	0.20 ± 0.04a	0.17 ± 0.02b	0.12 ± 0.01c
*P*_C+B_ (g g^−1^)	0.27 ± 0.05a	0.21 ± 0.03b	0.15 ± 0.02c
*P*_L_ (g g^−1^)	0.11 ± 0.021a	0.08 ± 0.009b	0.04 ± 0.007c
*P*_T_ (g g^−1^)	0.39 ± 0.05a	0.28 ± 0.04b	0.19 ± 0.03c
*N*_B_ (g m^−2^)	0.08 ± 0.006a	0.07 ± 0.007a	0.04 ± 0.006b
*N*_C_ (g m^−2^)	0.28 ± 0.04a	0.25 ± 0.03a	0.21 ± 0.03b
*N*_C+B_ (g m^−2^)	0.36 ± 0.06a	0.32 ± 0.04a	0.25 ± 0.04b
*N*_L_ (g m^−2^)	0.17 ± 0.02a	0.11 ± 0.02b	0.07 ± 0.01c
*N*_P_ (g m^−2^)	0.52 ± 0.06a	0.44 ± 0.06b	0.34 ± 0.05c
*N*_B_/*N*_P_	0.16 ± 0.03	0.14 ± 0.01	0.15 ± 0.02
*N*_C_/*N*_P_	0.51 ± 0.07b	0.63 ± 0.06a	0.64 ± 0.09a
*N*_L_/*N*_P_	0.32 ± 0.04a	0.29 ± 0.04b	0.21 ± 0.03c
*N*_M_ (mg g^−1^)	22.4 ± 3.1a	19.8 ± 2.3b	21.6 ± 2.7a
Chl (mg g^−1^ fw)	1.66 ± 0.21b	1.80 ± 0.25b	2.17 ± 0.17a
G_s_ (mol m^−2^ s^−1^)	0.26 ± 0.07a	0.22 ± 0.08b	0.13 ± 0.05c
C_i_ (μmol mol^−1^)	248.9 ± 22.4c	257.8 ± 16.3b	276.5 ± 21.7a
*P*_max_/*N*_P_ (μmol g^−1^ s^−1^)	27.12 ± 5.3a	20.82 ± 5.2b	16.91 ± 4.6c

*Significant differences were marked by different letters among the successional forests at the p = 0.05 level. Differences were analyzed using a Student–Newman–Keuls test*.

In contrast, the mean leaf *N*_A_ of the dominant species increased from the early- to the late-succession forests, with the lowest values being recorded in PF (Figure 2D). Although late-successional forests had significantly higher *N*_*A*_ than the dominant species in the PF, the dominant species in both MF and BF did not notably differ.

In the late-successional stage, dominant species had the lowest *P*_C_, *P*_C+B_, *P*_L_, *P*_T_, *N*_B_, *N*_C_, *N*_B+C_, *N*_L_, and *N*_P_ values when comparing PF and MF (Table 2). However, there were no significant differences for *N*_B_, *N*_C_, and *N*_B+C_ between PF and MF. These results showed that the dominant species decreased their photosynthetic capacity when the forest succeeded to the late, or relatively stable, stage (BF) in comparison to early- and mid-stage forests (PF and MF).

### Relationships between the leaf traits

**Figure 3:**
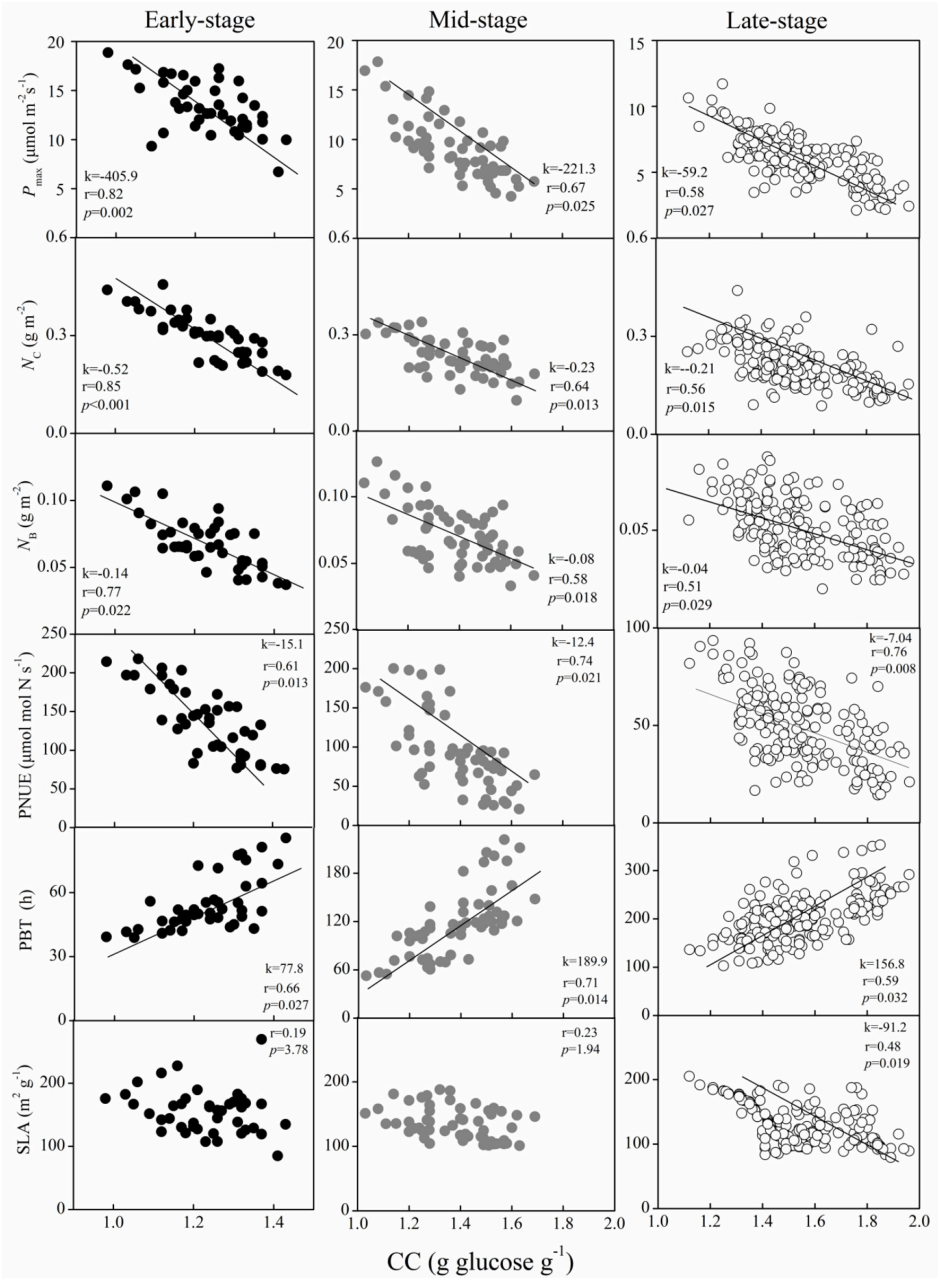
Relationship between the light saturated photosynthetic rate (*P*_max_), leaf nitrogen allocated to carboxylation (*N*_C_), bioenergetics (*N*_B_), photosynthetic N use efficiency (PNUE), payback time (PBT), and specific leaf area (SLA) with leaf construction cost (CC) in dominant species at the early-, mid-, and late-successional forests in sub-tropical China. Black, gray, and white circles indicate species from early-, mid-, and late-successional stages, respectively.

In each successional stage, some of the leaf functional traits showed significant relationships with leaf CC. For instance, the *P*_*max*_, *N*_B_, *N*_C_, and PNUE were negatively correlated with leaf CC, whereas PBT was positively correlated (Figure 3). Surprisingly, the significant relationships between SLA and leaf CC were only found in BF (Figure 3). Interestingly, the relationship between the *P*_*max*_ and leaf CC of the dominant species strengthened from the early- to the late-succession forests, as indicated by an increase in the correlation coefficient (*r*-values). In comparison, the strength of the relationship for *N*_B_, *N*_C_, and PNUE with the leaf CC of the dominant species weakened from PF to BF, based on the decrease in the *r*-values (Figure 3). The shift in this linkage implied that dominant species in the early-succession forest depress the light saturated photosynthetic rate, while investing more resources (e.g., N) in carboxylation and bioenergetics, and maintaining higher photosynthetic N use efficiency for “fast” growth.

**Figure 4:**
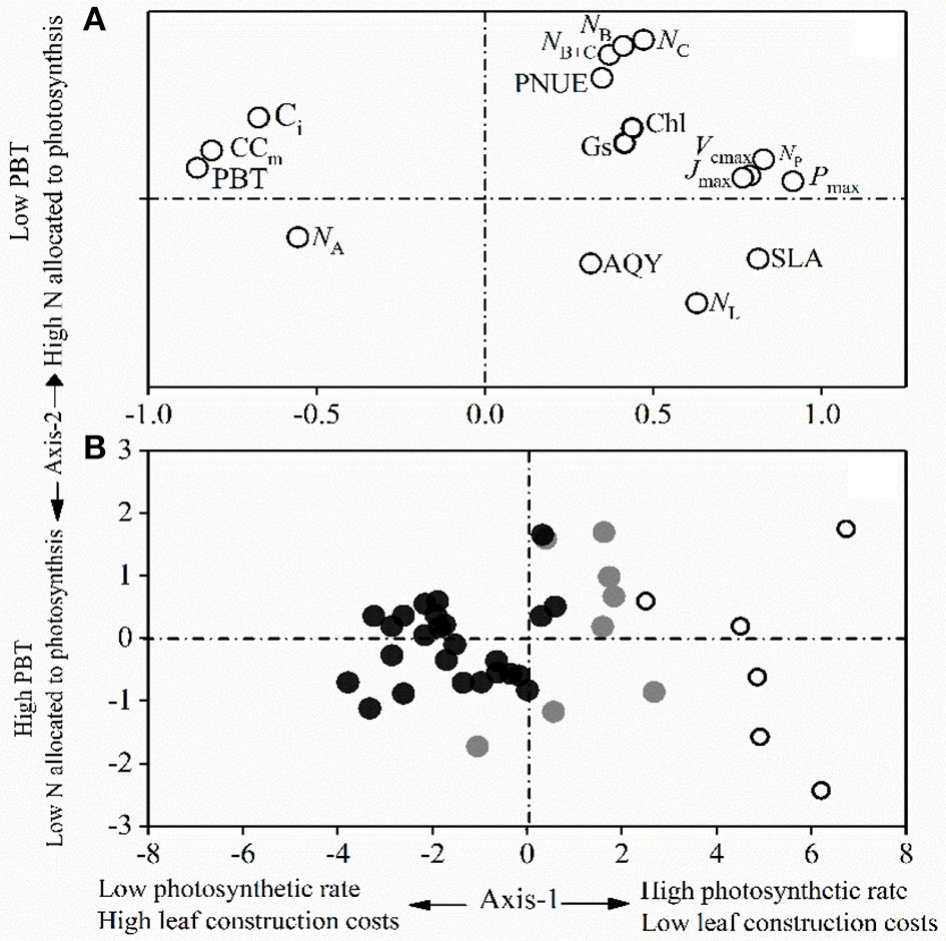
Principal component analysis for **(A)** the 17 leaf traits and **(B)** the studied component species for the first two axes. White, gray, and black circles indicate species from early-, mid-, and late-succession forests, respectively.

The principal component analysis on the 15 tested traits of the dominant species captured 73.21% of the variance in two principal components, with 50.53% being explained by the first axis and 22.68% being explained by the second axis (Figure 4). The first axis correlated positively with *P*_max_, *J*_max_, *V*_cmax_, SLA, and PNUE, and negatively with leaf CC. This difference represented a contrast between the “fast-growing” and “slow-growing” strategies of different plant species. Leaf N allocation to photosynthesis content (*N*_B_, *N*_C_, and *N*_B+C_) was positively and negatively correlated with the PBT loads of the PCA second axis (Figure 4). The dominant species from each forest succession were well separated along the first PCA axis. In comparison to the dominant species in the late-succession forest (BF), species in PF had the highest maximum CO_2_ assimilation rate (*P*_max_), SLA, *N*_B_, *N*_C_, and PNUE, but the lowest leaf CC and PBT.

## Discussion

Resource investment is one of the fundamental strategies for plant growth, survival, and productivity (Prach and Walker, 2011). Supporting previous studies (Guariguata and Ostertag, 2001; Poorter and Bongers, 2006; Chai et al., 2015), our results showed that the mean leaf CC and the PBT of the dominant species significantly increased from the early to the late successional stages (from PF to BF) despite there existed significant differences in some of the species in each forest (Figure 1). The mean leaf CC of the dominant species in the early succession (1.21 ± 0.04 g glucose g^−1^) and late succession (1.56 ± 0.07 g glucose g^−1^) forests in this region was lower and higher, respectively, than those recorded in the tropical forests of southwestern China (1.38 ± 0.01 g glucose g^−1^, Zhu and Cao, 2010) and those in Brazilian tropical forests (~1.20 g glucose g^−1^; Falcão et al., 2015); however, both the leaf CC and PBT in this study indicated that dominant species actively adjusts their investment in the leaves to balance cost-benefits during succession by shifting the composition of dominant species (Table 1). In the early successional forest (PF), the dominant species, such as *P. massoniana, C. fissa*, and *E. lepta*, had higher *P*_max_ (14.0 ± 3.2 μmol m^−2^ s^−1^) and SLA (163.2 ± 17.5 cm^2^ g^−1^) than the dominant species in the late successional forest (BF), with 6.1 ± 1.5 μmol m^−2^ s^−1^ *P*_max_ and 121.7 ± 13.2 cm^2^ g^−1^ SLA, respectively (Figure 2). This difference might represent an adaptive strategy. For instance, shortening the PBT and lowing the leaf CC maintains the “fast growing” for early successional species (Navas et al., 2003; Pýankov et al., 2011; Raevel et al., 2012; Reich, 2014; Chai et al., 2016).

The higher gas exchange rates (shown by *P*_max_, *J*_max_, and *V*_cmax_; Table 2), but lower leaf CC (Figure 2), in PF versus BF further implied that the dominant species in the early-successional forest assimilate large amounts of energy crucial for tissue construction *via* photosynthesis in a relatively shorter time. This strategy leaves more energy for growth and quick-return leaves (e.g., lower PBT; Poorter and Bongers, 2006; Feng et al., 2008). This strategy benefits the survival of pioneer species, providing them with the opportunity to construct a community. With the closing of the canopy in the mid-successional forest, the dominant species were replaced with *C. chinensis, S. superba, A. quinquegona, S. heptaphylla*, and *P. asiatica* (Table 1). These species are characterized with lower gas exchange rates (6.1 ± 1.5 μmol m^−2^ s^−1^ *P*_max_, 75.6 ± 8.8 μmol m^−2^ s^−1^ *J*_max_, and 36.3 ± 3.7 μmol m^−2^ s^−1^ *V*_cmax_, Table 2). This type of change helps the dominant species enhance their shade tolerance. In the BF, the much lower *P*_max_, PNUE, and SLA (Figure 2, Table 2) reflected that the dominant species must invest more in assimilating biomass to survive, to avoid growing in closed-canopy (shaded) environments with limited resources (Poorter, 2009; Chai et al., 2015).

Usually, tree species with higher SLA have a larger ability to capture available resources, because the SLA is closely related to the leaf benefit-cost ratio (Grotkopp and Rejmaánek, 2007). Several studies have shown that dominant species in early-successional forest have higher SLA than those in late-successional forests (Rijkers et al., 2000; Garnier et al., 2004; Navas et al., 2010; Chai et al., 2015). Notable variation in SLA among the succession forests in the current study (Figure 2) showed that species in the early succession forest adaptively act with higher photosynthesis, resource utilization efficiency, and faster growth rates to establish the community when compared to species in the late successional forest. The decreased photosynthetic capacity of species in the late successional forest was indicated by it having the lowest *P*_C_, *P*_C+B_, *P*_L_, *P*_T_, *N*_B_, *N*_C_, *N*_B+C_, *N*_L_, and *N*_P_ (Table 2). This result again confirmed that species are able to shift their photosynthetic characteristics and leaf area actively with forest succession.

Among the successions, only the dominant species in the late stage had negatively significant relationships between SLA and leaf CC (Figure 3). This result supported the finding that species in the late successional forest had the smallest SLA (Figure 2), but the highest leaf CC (Figure 1). This negative significance implies that species should invest more to access resources under the closed canopy during late succession. The lack of significant correlation between the SLA and leaf CC of species in the earlier successional forests might be due to large variations in SLA across species in the fragmented environment and among species within the same open site (Bassow and Bazzaz, 1997; Jacobsen et al., 2007).

The PNUE is an important leaf trait that characterizes species in relation to their leaf economics, physiology, and adaptive strategies (Onoda et al., 2017), and might indirectly reflect the efficiency of N utilization (Feng et al., 2008). The notable higher PNUE of the dominant species in the early-successional forest compared to the later succession stage (Figure 2) suggested that the utilization N efficiency of photosynthesis decreased from the early to the late successional forest. Similar results have been obtained in the forests of the Amazon and temperate forests (Reich et al., 1995; Chai et al., 2015). The weakening of the relationships between *P*_max_ and the leaf CC were indicated by the decreasing *r* values from the early to the late successional forests in our study (from 0.82 to 0.58, Figure 3), which further confirmed that species in the late successional stage have invariably low leaf investment.

Species with high PNUE might invest a substantial amount of N to carboxylation and bioenergetics to increase the potential of carbon gain (Feng et al., 2008). In this study, we found that the dominant species in the early-, mid-, and late-succession forests presented notably different nitrogen allocation to carboxylation, bioenergetics, and light-harvesting components as well as stomatal conductance (Table 2). When significantly higher fractions of photosynthetic N were allocated to bioenergetics and carboxylation (*P*_C_), the *N*_B_, *N*_C_, *N*_C+B_, light-harvesting component (*P*_L_), *N*_L_, and *N*_P_ of species in the early- and mid-successional forests were higher than in the late-succession forest. This difference showed that species in the early- and mid-succession forests had relatively higher PNUE than those in the late-succession forest. This difference might due to the higher efficiency of photosynthetic N partitioning, especially for high *N*_C_, in the early- and mid-succession stages (Feng et al., 2008; Zhu et al., 2012), since C_i_ did not decrease by the lower G_s_ in the late successional forest. Thus, C_i_ may not be a major factor determining the differences in *P*_max_ and PNUE among forest types in this study (Table 2). The weakening relationship for *N*_C_ and *N*_B_ with leaf CC from PF to BF (*r* values decreased from 0.85 to 0.56 for *N*_C_ and from 0.77 to 0.51 for *N*_B_, Figure 3) in this study, and previous research (Feng et al., 2008; Onoda et al., 2017), confirmed that species in the early successional stage actively enhance their investment in resources by increasing their allocation of leaf N invested in photosynthesis to adapt to the environment.

Plants with a “slow-growing” strategy are associated with a low light-saturated photosynthetic rate (*P*_max_) and return per unit time on investment in leaf nutrients (e.g., PNUE and leaf N allocation to Rubisco; Onoda et al., 2017), but with a higher proportional allocation of structural N and longer payback time, as shown in late-successional species (Chai et al., 2015; Reich and Flores-Moreno, 2017). Alternatively, “fast-growing” strategy species with higher *P*_max_ tend to have higher PNUE and a larger fraction of leaf N allocated to photosynthesis (Wright et al., 2004; Feng et al., 2008; Hikosaka, 2014; Onoda et al., 2017). As well known, stomatal closure decreases photosynthetic rate through a decrease in C_i_, leading to a decrease in PNUE (Zhu et al., 2012). The significant decrease of *N*_B_, *N*_C_, and the mean stomatal conductance (G_s_, Table 2) and the relationships between G_s_ and *P*_max_ (*r*-values decreased from 0.84 to 0.64, Appendix Figure 1) from the early to the late successional stages implied that plants from early-succession stages had larger fraction of organic N being allocated to the photosynthetic apparatus in “fast-growing” species. Additionally, the significantly negative relationships between the mean *P*_max_ and C_i_ (*r*-values are −0.51, −0.72, and −0.35 for PF, MF, and BF, respectively, Appendix Figure 1) could indirectly reflect that the efficiency to use intercellular CO_2_ (associated with N in photosynthesis) was not dominantly important in explaining the higher *P*_max_ of the “fast-growing” species. The PCA results of the current study (Figure 4) further strengthened the concept of the leaf construction cost investment strategy and other associated leaf functional traits. Axis 1 revealed the tradeoff between photosynthetic nutrients and leaf dry mass per unit area (Figure 4), as reported by Wright et al. (2004) and Onoda et al. (2017), with a tradeoff between mesophyll CO_2_ diffusion and the fraction of leaf N in the cell wall (Reich, 2014; Onoda et al., 2017). The PCA analysis showed that the dominant species in the early-successional forest exhibited typical “fast-growing” characteristics, such as higher G_s_, *P*_max_, *J*_max_, *V*_cmax_, PNUE, *N*_B_, and *N*_C_, in addition to lower leaf CC and PBT. In comparison, species in the late-successional forest had lower *P*_max_, PNUE, and allocation of leaf N to photosynthesis. The PCA results further support that the dominant species in the early successional forest had a higher ability to capture resources, with a lower investment in “fast growing” adaptive strategies.

## Conclusions

The comparison of leaf functional traits for the dominant species from early-, mid-, and late-succession forests in subtropical China showed that the early-successional forest is characterized with significantly lower leaf construction costs, payback time, and leaf N content based on leaf area, but higher *P*_max_, special leaf area, photosynthetic N use efficiency, and leaf N allocated to carboxylation and bioenergetics, than the late-successional forest. The strengthening of the relationship between *P*_max_ and leaf CC, and the weakening of the relationship for *N*_B_, *N*_C_, PNUE, with leaf CC, from early- to late-successional forests, revealed that the dominant species actively decrease the allocation of the photosynthetic N fraction to carboxylation and bioenergetics as adaptive strategies during forest succession. The shifts in these leaf functional traits and their linkages might represent a fundamental physiological mechanism of forest succession and stabilization.

## Author contributions

YX and SL: Designed the research; YX and FT: Completed the field work; YX, SL, YK, and BC: Analyzed the data; YX, YK, and FT: Wrote the paper.

### Conflict of interest statement

The authors declare that the research was conducted in the absence of any commercial or financial relationships that could be construed as a potential conflict of interest.
