# Supplementary material for: Dominant Species in Subtropical Forests Could Decrease Photosynthetic N Allocation to Carboxylation and Bioenergetics and Enhance Leaf Construction Costs during Forest Succession
Source: Front Plant Sci. 2018 Feb 8;9:117. doi: 10.3389/fpls.2018.00117 (PMC5809493; doi:10.3389/fpls.2018.00117)
Supplement: Supplementary file 1 [file DataSheet1.docx]

**Appendix Figure 1**





**Appendix Figure 1** Light-saturated photosynthetic rate (*P*_max_) as a function of intercellular concentration of CO_2_ (C_i_) (a-c) and stomatal conductance (Gs) (d-f); C_i_ as a function of G_s_ (g-i) in dominant species at the early-, mid-, and late-successional forests in sub-tropical China. Black, grey, and white circles indicate species from early-, mid-, and late-successional stages, respectively.
